# Ubiquitin Ligase Redundancy and Nuclear-Cytoplasmic Localization in Yeast Protein Quality Control

**DOI:** 10.3390/biom11121821

**Published:** 2021-12-03

**Authors:** Carolyn Allain Breckel, Mark Hochstrasser

**Affiliations:** Department of Molecular Biophysics and Biochemistry, Yale University, New Haven, CT 06520, USA; carolyn.allain@yale.edu

**Keywords:** protein degradation, proteasome, ubiquitin, degron, protein quality control, ubiquitin ligase

## Abstract

The diverse functions of proteins depend on their proper three-dimensional folding and assembly. Misfolded cellular proteins can potentially harm cells by forming aggregates in their resident compartments that can interfere with vital cellular processes or sequester important factors. Protein quality control (PQC) pathways are responsible for the repair or destruction of these abnormal proteins. Most commonly, the ubiquitin-proteasome system (UPS) is employed to recognize and degrade those proteins that cannot be refolded by molecular chaperones. Misfolded substrates are ubiquitylated by a subset of ubiquitin ligases (also called E3s) that operate in different cellular compartments. Recent research in *Saccharomyces cerevisiae* has shown that the most prominent ligases mediating cytoplasmic and nuclear PQC have overlapping yet distinct substrate specificities. Many substrates have been characterized that can be targeted by more than one ubiquitin ligase depending on their localization, and cytoplasmic PQC substrates can be directed to the nucleus for ubiquitylation and degradation. Here, we review some of the major yeast PQC ubiquitin ligases operating in the nucleus and cytoplasm, as well as current evidence indicating how these ligases can often function redundantly toward substrates in these compartments.

## 1. Introduction

Cells depend on the proper maintenance of protein homeostasis, including regulation of protein levels in response to external stimuli or during development and clearance of damaged or dysfunctional proteins. Genomic mutations or errors during transcription or translation can produce erroneously folded proteins that are incapable of carrying out their functions, and various chemical and physical stressors can similarly induce protein misfolding [1]. Misfolded proteins can be found in all compartments of the cell, and therefore, diverse pathways to handle these proteins are necessary in each. In eukaryotes, misfolded proteins are managed by various protein quality control (PQC) systems that repair or degrade them. When abnormal proteins cannot be refolded, their destruction is accomplished in large part by the actions of the ubiquitin-proteasome system (UPS) [2]. Such aberrant proteins often form intracellular aggregates that can be toxic to cells if they are not cleared in a timely fashion. Importantly, several neurodegenerative diseases and protein misfolding diseases—termed proteinopathies—appear to be connected to this protein aggregation phenomenon in human cells, including Alzheimer’s disease, Parkinson’s disease, and Huntington’s disease [3]. PQC is, therefore, vital to cell health, but the exact mechanisms by which misfolded proteins are recognized and directed to the diverse pathways of the UPS remain to be resolved.

## 2. The Ubiquitin-Proteasome System in *Saccharomyces cerevisiae*

**Figure 1:**
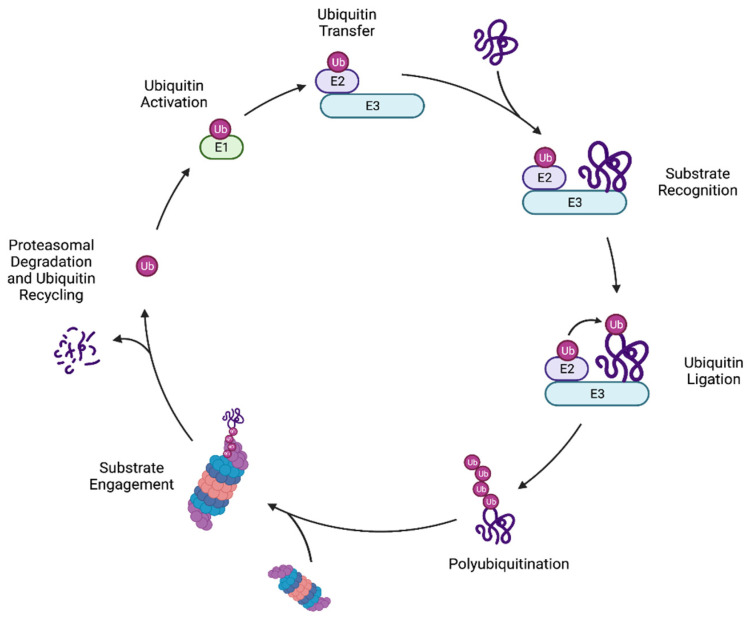
The ubiquitin-proteasome system. Ubiquitin is activated by and covalently binds a ubiquitin-activating enzyme (E1) and is transferred to a ubiquitin-conjugating enzyme (E2). A ubiquitin ligase (E3) recognizes a protein substrate and simultaneously binds the E2-Ub conjugate. The E3 enzyme mediates transfer of the ubiquitin to a substrate protein. Multiple rounds of ubiquitylation can be performed on a given substrate to create polyubiquitin chains. Ubiquitylated substrates are recognized by the proteasome. Substrates are deubiquitylated, unfolded, and translocated into the proteasome core for degradation. Substrate peptides are released, and intact ubiquitin molecules are recycled.

Regulation of protein homeostasis (“proteostasis”) is mediated in part by molecular chaperones, which assist in the establishment and maintenance of appropriate protein conformations, and the UPS, which degrades aberrant proteins which cannot be refolded. Protein substrates are marked for destruction by the 26S proteasome through covalent attachment of the conserved ubiquitin protein [2,4]. Ubiquitylation of a protein is carried out by a cascade of enzymes: a ubiquitin-activating enzyme (E1), one or more ubiquitin-conjugating enzymes (E2), and one or more ubiquitin ligases (E3) (Figure 1). The E1 first forms a high-energy thioester bond between the ubiquitin C-terminus and its own active site cysteine residue in an ATP-dependent manner. This activated ubiquitin is then transferred to an E2 cysteine side chain via a transthiolation reaction. Finally, an E3 enzyme binds both the protein substrate and the E2-Ub conjugate and promotes the transfer of ubiquitin to a substrate lysine residue [5,6]. In yeast, there is one ubiquitin-activating enzyme (Uba1) along with 11 ubiquitin-conjugating enzymes, and roughly 100 ubiquitin ligases, reflecting the wide range of substrates that must be recognized by E3 enzymes, often for specific proteasome-mediated degradation [7,8]. The mechanisms by which ubiquitin ligases recognize misfolded proteins for degradation are closely tied to the type of degradation signal or “degron” that is displayed by the substrate protein [2,7].

A target protein can be ubiquitylated at a single residue (mono-ubiquitylation), mono-ubiquitylated at several residues (multi-ubiquitylation), or have a chain of ubiquitin moieties appended to a single protein site (poly-ubiquitylation) [9]. Although protein lysyl ubiquitylation is most common, it is also possible for serine, threonine, cysteine, or the substrate N-terminal methionine amino group to be used as ubiquitin attachment sites [10]. There is considerable variability in the possible poly-ubiquitin chains that can be formed. A ubiquitin moiety can be covalently attached to one of the seven lysine residues on another ubiquitin molecule (K6, K11, K27, K29, K33, K48, and K63), producing unbranched (homotypic) or branched (heterotypic) poly-ubiquitin chains [4]. An unbranched poly-ubiquitin chain is composed of a single linkage type, while a branched poly-ubiquitin chain can contain several different amide (isopeptide) linkages [11]. Protein ubiquitylation can have various consequences depending on the ubiquitin configuration on the protein substrate. Protein mono-ubiquitylation has been associated with many processes, including DNA repair, autophagy, and membrane trafficking. Poly-ubiquitylation often directs protein substrates to the proteasome [12,13]. Polyubiquitin chains of various linkages can mark substrates for destruction, but the majority of proteasome-mediated degradation is mediated by K48 and also K11 ubiquitin chains [7].

An important facet of ubiquitylation is that it is readily reversible due to the action of de-ubiquitylating enzymes (DUBs), of which there are at least 21 in budding yeast [14,15]. The yeast proteasome subunit Rpn11, for example, is a DUB that removes ubiquitin chains from proteasome-bound substrates, facilitating subsequent ATP-dependent substrate unfolding by the proteasomal AAA+ ATPase ring [16]. This leads to degradation of the protein and recycling of the ubiquitin moieties (Figure 1) [17].

In the case of large protein aggregates or damaged organelles, the cell can instead degrade these substrates through autophagy. The macroautophagy pathway encloses substrates in double-membrane sacs called autophagosomes that subsequently fuse with the vacuole (mammalian lysosome) where their contents are digested. Autophagy is typically induced under stress conditions such as starvation [18,19]. Under starvation, autophagosomes usually engulf random volumes of cytoplasm; however, autophagy can be selective, and such mechanisms often also employ ubiquitin as a specificity tag [18,19]. This review will focus on soluble substrate recognition and ubiquitylation by ubiquitin ligases that lead to substrate degradation by the proteasome.

Budding yeast proteasomes reside primarily in the cell nucleus (~80%), and this localization is generally evolutionarily conserved [20,21,22]. In the fission yeast *Schizosaccharomyces pombe* and the green alga *Chlamydomonas reinhardtii*, proteasomes concentrate at the inner nuclear envelope; mammalian proteasome localization, while often also mostly nuclear, varies among different cell types [20,21,22,23,24,25]. The need for the concentration of proteasomes in the nucleus is not well understood. One possibility is the high number of transcription factors present in the nucleus that must be rapidly turned over, and the important contributions of the UPS to DNA replication and repair [26]. Another possibility is that misfolded proteins in the nucleus are a greater threat than in other compartments. Studies in mammalian cells have shown that misfolded protein aggregates can be cytotoxic to cells by sequestering their interaction partners. These can include proteasome subunits, chaperone and co-chaperone proteins, transcription factors, and RNA [27]. The functions of these species can be compromised, and nuclear processes may be particularly sensitive to these deficits. Despite the concentration of proteasomes in the nucleus, dysfunctional proteins can arise in all parts of the cell, necessitating diverse recognition pathways.

**Figure 2:**
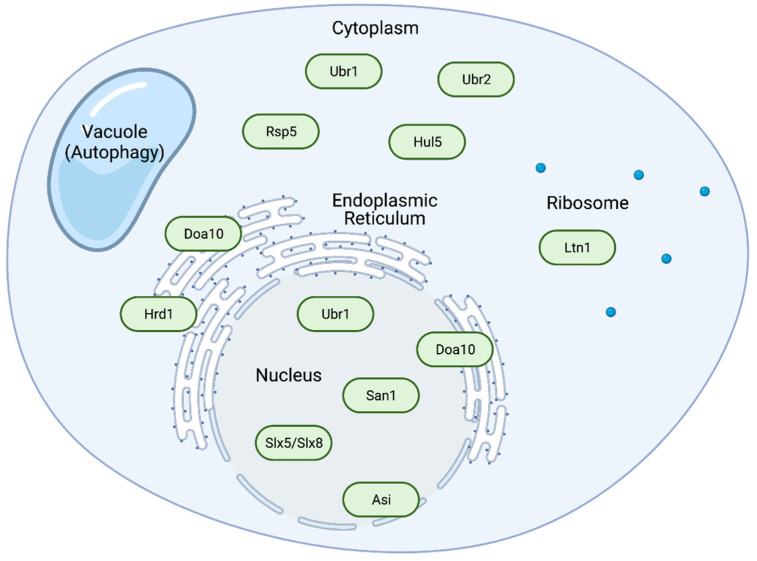
Localization of the major yeast ubiquitin ligases. Diagram of a yeast cell depicting the major sites of protein quality control and the associated ubiquitin ligases (green). Misfolded substrates can be recognized by different ubiquitin ligases in different compartments for efficient degradation. The vacuole can mediate destruction of larger aggregates through the process of autophagy.

Misfolded or otherwise dysfunctional proteins must be distinguished from their properly folded counterparts and nascent proteins that have not yet fully folded or reached their proper subcellular destinations. Target proteins are typically ubiquitylated by ubiquitin ligases in a compartment-specific manner, with distinct ubiquitin ligases operating in the nucleus and cytoplasm, and at organelles such as the endoplasmic reticulum (ER) (Figure 2, Table 1) [6]. The ER is an especially important site for protein degradation, where it is termed ER-associated degradation (ERAD). Due to extensive ‘de novo’ protein folding, processing, and modification in the ER lumen and bilayer, ERAD is especially important for quality control, where ER-resident ubiquitin ligases must distinguish folding nascent proteins from those that have become terminally misfolded or misassembled [6,28]. The ER membrane is continuous with the outer membrane of the nuclear envelope (NE), and the inner and outer membranes of the NE fuse at nuclear pore complexes (NPCs) [29]. Different ER-associated ubiquitin ligases in this functionally subdivided ER recognize substrates in the cytoplasm, nucleus, or ER, depending on whether the substrate is soluble and outside the ER, luminal, or membrane-embedded [28].

Ubiquitin-dependent proteolytic targeting of misfolded proteins in the nucleus and cytoplasm is less understood than that of substrates in the ER itself. Unlike such ERAD substrates, it is unclear whether these substrates are ubiquitylated in their resident compartment or first trafficked to a different cellular site. Several studies have indicated redundancy between the function of several E3 enzymes in the nucleus and cytoplasm as well as an apparent requirement for translocation of certain substrates between compartments for efficient clearance [30,31,32,33]. This review will focus on the model eukaryote *Saccharomyces cerevisiae*, where many of the fundamental discoveries about ERAD and other PQC degradation pathways have been made, and detail how the primary PQC ubiquitin ligases operate within the nucleus and cytoplasm and how they overlap in their compartment-specific substrate recognition properties.

## 3. Degradation of Nuclear Substrates

The nucleus houses the genome and crucial machinery for the expression, replication, and safeguarding of the genome; it is also the site for ribosome biogenesis and other complex RNP assembly processes [34]. As speculated above, these attributes might help explain the high concentration of proteasomes in the nucleus as these processes demand a high level of PQC and protein degradation. The nucleus is partitioned from the cytoplasm by the NE, only permitting carrier-mediated macromolecular movement or passive diffusion of sufficiently small proteins and other molecules through NPCs [35,36]. In yeast, the NE does not break down during mitosis—unlike in most metazoans—so additional nucleus-specific factors might be required for yeast nuclear PQC, here referred to as NucPQC, since access of the cytoplasmic PQC machinery to the nucleus could be limited [37]. NucPQC ensures that resident aberrant proteins and cytoplasmic substrates entering the nucleus through “leaky” NPCs are rapidly turned over [20,38,39].

Recent reports indicate that nascent 26S proteasomes are imported into the yeast nucleus following their assembly in the cytoplasm and that proteasomal nuclear localization is essential in yeast [22,33,40]. Their nuclear accumulation notwithstanding, some have questioned whether 26S proteasomes are catalytically active within yeast or mammalian nuclei [41]. Despite this uncertainty, nuclear-localized E3 enzymes are known to be necessary for ubiquitylation of misfolded and naturally short-lived nuclear proteins, targeting them for degradation by catalytically active proteasomes—wherever these may reside [42].

No protein synthesis is believed to occur in the cell nucleus, so the NucPQC machinery does not typically need to recognize misfolded nascent polypeptides. Nuclear ubiquitin ligases target conformationally unstable or disordered proteins as well as nuclear proteins that are damaged or misfold due to environmental insults or cell stressors [34]. Inefficient turnover of misfolded nuclear proteins can often result in protein aggregates known as intranuclear quality control (INQ) compartments [43,44]. INQ aggregates have been characterized as dedicated compartments that sequester misfolded nuclear proteins until they are disaggregated and refolded or degraded by limiting NucPQC components and molecular chaperones. Refolding of aggregated INQ proteins usually occurs in a manner dependent on the combined action of the Hsp70, Hsp40, and Hsp104 molecular chaperones; the Hsp104 disaggregase (human CLPB), an AAA+ ATPase, extracts misfolded proteins from aggregates and these proteins are then refolded by the Hsp70 chaperone and the Hsp40 co-chaperone Sis1 [45]. However, the Hsp40 protein Apj1, along with Hsp70 and the nucleotide-exchange factor Hsp110, disaggregates misfolded nuclear substrates and targets them for degradation instead [45]. Interestingly, INQ compartments contain both nuclear and cytoplasmic misfolded substrates, suggesting that stress conditions favor nuclear turnover of cytosolic proteins [45]. Failure of the UPS to clear nuclear aggregates can eventually become toxic to cells and may lead to various neurodegenerative diseases in humans [46]. Nuclear-resident ubiquitin ligases may have evolved to recognize distinct features of misfolded proteins in the nucleus and help limit the formation of long-lived toxic nuclear aggregates.

### 3.1. The San1 Pathway

San1 is a nuclear localized ubiquitin ligase; no mammalian homolog has been identified and it may be restricted to yeast species (Table 1) [47,48]. San1 contains a bipartite nuclear localization signal (NLS) and requires nuclear accumulation to function [47]. It is also a mostly intrinsically disordered protein that appears to lack a defined structure outside of the catalytic RING domain, which activates ubiquitin transfer from its cognate E2s to protein substrates [49]. Operating primarily with the E2 Ubc1, San1 recognizes a “window of hydrophobicity” in a target protein, usually a sequence of at least five consecutive hydrophobic amino acids [47,50]. Proteins with fewer than five exposed hydrophobic amino acids are not degraded in a San1-dependent manner, despite being highly hydrophobic overall. San1 recognition is therefore specific for locally concentrated hydrophobicity (typically valine, leucine, and isoleucine residues), rather than overall protein hydrophobicity [50]. Additionally, early studies into San1 specificity demonstrated that it recognizes unfolded mutant proteins but does not ubiquitylate their normal counterparts, underscoring its dedicated role in the quality control of nuclear misfolded proteins [47].

The disordered regions of San1 are indispensable for substrate recognition; these regions appear to contain multiple short substrate-binding sites that associate with target proteins. San1 recognition of unfolded proteins is direct, though the exact mechanism by which its substrate-binding sites interact with its targets remains unclear [49]. Two models have been proposed to account for how San1 may bind its substrates with unstructured domains. The first suggests that San1 grasps an unfolded substrate by making simultaneous contacts through many of its binding sites; the second model proposes that each San1 substrate binding site operates independently of the others, each forming weak interactions with substrates but affording San1 greater avidity for its diverse targets [49]. In either model, the flexibility of the San1 disordered domains and their many binding sites allow the E3 to accommodate a wide variety of misfolded substrates. Notably, San1 does not interact nonspecifically with all intrinsically disordered proteins, confirming its specificity for certain misfolded targets [50]. San1 also does not ubiquitylate itself significantly despite its own disordered structure; this is due to the paucity of lysine residues in these domains that could serve as modification sites [51].

**Figure 3:**
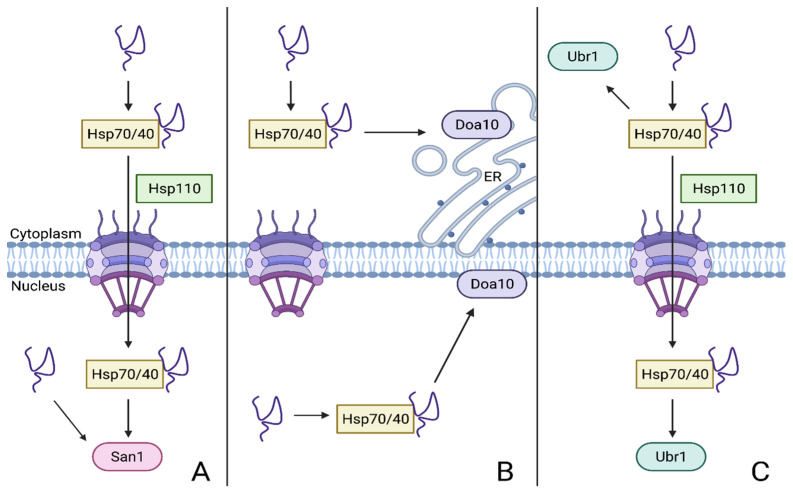
Chaperone-dependent substrate nuclear translocation and recognition by PQC ubiquitin ligases. (**A**) San1-mediated ubiquitylation. San1 is a nuclear ubiquitin ligase (E3) that directly recognizes hydrophobic residues in misfolded proteins. In some cases, misfolded cytoplasmic proteins are transported to the nucleus for San1-mediated ubiquitylation. This process requires Hsp70, Hsp40, and Hsp110 chaperone proteins, and San1 is redundant with Ubr1 and Doa10 in some cases. (**B**) Doa10-mediated ubiquitylation. Doa10 is an ER/NE-associated ubiquitin ligase that can localize to the cytoplasmic and nuclear faces of the NE. Doa10 functions in ERAD-C for degradation of soluble and membrane proteins at the ER. Doa10 substrate recognition typically requires Hsp70 and Hsp40 chaperone proteins, possibly to maintain substrate solubility prior to ubiquitylation. (**C**) Ubr1-mediated ubiquitylation. Ubr1 is a predominantly cytoplasmic E3 that directly recognizes cytoplasmic misfolded proteins in the N-degron pathway. Independent of this pathway, Ubr1 can also localize to the nucleus and ubiquitylate misfolded cytoplasmic proteins after they have been translocated into the nucleus by a molecular chaperone-dependent mechanism. This mode of E3 substrate targeting can be redundant with San1 and Doa10.

**Figure 4:**
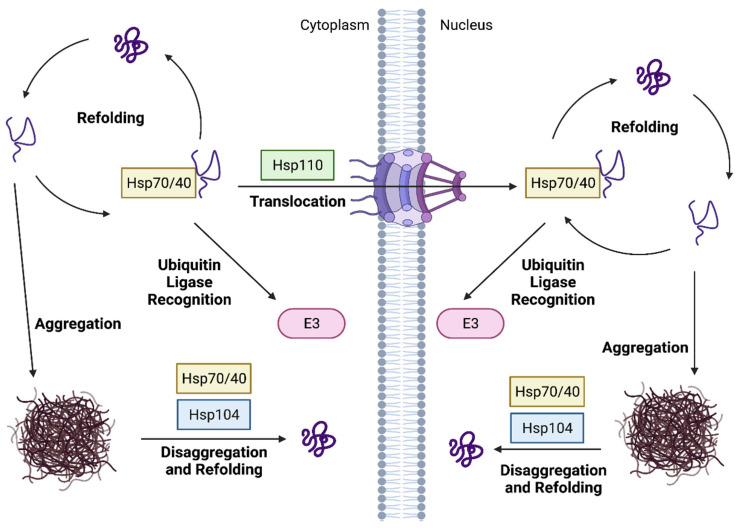
The roles of chaperone proteins in cellular proteostasis. Chaperone proteins play a major role in protein quality control. Misfolded proteins in either the nucleus or the cytoplasm can be refolded by the action of Hsp70 chaperones, Hsp40 co-chaperones and Hsp110 nucleotide-exchange factors. If refolding is not possible, these molecular chaperones can mediate transport of a substrate to ubiquitin ligases for substrate recognition and ubiquitylation. Hsp70/40/110 chaperones likely maintain solubility of misfolded proteins prior to ubiquitin ligase recognition. For certain cytoplasmic substrates, nuclear import is required for ubiquitylation, although the mechanism of import is not known. If misfolded proteins are not quickly refolded or degraded, they can aggregate in various cellular compartments. In some cases, the chaperone Hsp104 contributes to disaggregation and Hsp70/40-mediated protein refolding.

It is likely that San1 senses unfolded proteins by recognizing exposed hydrophobic residues that would normally be buried in the target protein core. Many of the identified San1 substrates were also shown to aggregate in the nucleus and induce cytotoxicity in yeast; importantly, San1 is unable to recognize and ubiquitylate proteins if they have aggregated, likely because it recognizes the hydrophobic residues that become buried within aggregate structures [49]. While molecular chaperones are not required for San1 to bind its substrates, Hsp70/40/110 chaperones help translocate many cytoplasmic substrates to the nucleus for San1-mediated ubiquitylation (discussed in Section 5.2). These chaperones may also maintain the solubility of disordered proteins so that they do not aggregate and evade San1 detection (Figure 3A and Figure 4) [31,34]. San1 deletion yields a chronic cellular stress response when cells are grown in minimal medium, but it does not stabilize ER-associated PQC substrates [47,48]. Altogether, these data suggest that San1 recognizes misfolded proteins through their exposed hydrophobicity before they have a chance to aggregate in the nucleus and cause toxicity.

### 3.2. The Doa10 Pathway

Another E3 enzyme that operates in the cell nucleus is Doa10. Doa10 is a large integral membrane protein that contains 14 transmembrane domains and an N-terminal RING domain, though a full structure of the protein has yet to be solved [52]. Unlike San1, Doa10 is broadly conserved in a wide range of eukaryotes, including humans (MARCH6F/TEB4). It requires two different ubiquitin-conjugating enzymes, Ubc6 and Ubc7, for its function [53,54]. Doa10 resides in all regions of the ER, including the inner nuclear membrane (INM), and participates in ERAD [28]. This broad distribution allows Doa10 to recognize soluble substrates in both the cytoplasm and nucleus as well as membrane proteins in the ER and NE (Figure 3B) [42]. The wide range of substrates that are recognized by Doa10 indicate that it is a central component of PQC throughout the cell [34]. Substrates of the ERAD pathways are mainly defined by the location of their degrons; the degrons can be within the ER lumen (ERAD-L), the membrane-spanning region (ERAD-M), or the cytoplasmic or nuclear domains (ERAD-C) of the target proteins [28]. Although Doa10 can participate in ERAD-M, this review will mainly focus on its role in soluble ERAD-C. Doa10 is the primary E3 responsible for clearance of ERAD-C substrates in yeast [42].

Previous studies into ERAD-C degrons have revealed unusual consistency in the degron characteristics typically recognized by Doa10. In particular, Doa10 targets proteins that contain an exposed amphipathic α-helical domain that is also quite hydrophobic. Perturbations in the helical hydrophobic sequences abrogate Doa10 recognition, but there does not appear to be any consensus sequence among the various identified Doa10 degrons. These observations suggest that Doa10 recognition most often requires a hydrophobic surface in the context of an α-helix, as opposed to general hydrophobicity or helicity per se [55,56,57,58,59,60]. Moreover, one study showed that a soluble ERAD substrate containing an amphipathic helix, which requires Doa10 for its degradation in yeast, could bind to membranes, and it is possible that other soluble Doa10 substrates may have some propensity to associate with membranes as well [61,62].

Exactly how Doa10 interacts with its substrates remains to be determined; the strong topological conservation of the E3 and the conservation of charged or polar residues in certain of its transmembrane helices suggest an association between degron-containing proteins and the Doa10 transmembrane domains, possibly during substrate extraction from the membrane [63]. Transmembrane substrates of Doa10 have been shown to require the Cdc48 AAA+ ATPase (mammalian p97) for their degradation, unlike soluble substrates, even those bearing the same degron [57]. This implicates Cdc48 specifically in protein retrotranslocation from the ER membrane of Doa10-modified proteins. Doa10 also has been directly implicated in the retrotranslocation process [63,64]. In addition, Doa10 may require chaperone proteins to select misfolded substrates or maintain them in a state that allows their ubiquitylation (Figure 3B) [28,34].

As mentioned earlier, Doa10 functions with two different E2 enzymes, Ubc6 and Ubc7, in substrate ubiquitylation, and both E2s are required for degradation of most Doa10 substrates [65]. Ubc6 is a tail-anchored membrane protein and primarily functions with Doa10 for attachment of mono-ubiquitin or short K11-linked ubiquitin chains, while Ubc7 specifically forms K48-linked chains [66]. Ubc7 is a soluble protein that requires the transmembrane activator protein Cue1 to help tether it to the ER membrane and mediate Ubc7 E2 activity [67,68,69]. Current studies favor a ubiquitylation model in which the two E2 enzymes act in a sequential manner. It is proposed that Ubc6 supplies an initial ubiquitin monomer to a substrate that is then elongated by Ubc7-mediated poly-ubiquitylation [70]. Although Ubc6 and Ubc7 were reported to interact with each other and both bind Doa10, it is not currently known how they physically interact with Doa10 [62].

### 3.3. Other Nuclear Recognition Pathways

The Asi complex is another INM-associated E3 ligase that participates in NucPQC. The complex comprises three integral membrane proteins—Asi1, Asi2, and Asi3—that function exclusively at the INM [71,72]. Asi1 and Asi3 each have a C-terminal RING domain and, like Doa10, have been shown to associate with the ubiquitin-conjugating enzymes Ubc6 and Ubc7, and weakly with Ubc4 [73]. It has been proposed that the Asi complex utilizes a sequential mechanism similar to that of Doa10, using Ubc4 and Ubc7 for ubiquitylation [74]. By contrast, Asi2 does not possess ligase activity and is dispensable for ubiquitylation of some Asi substrates. Asi2 may serve as an adaptor or regulator of substrate recognition, specifically of transmembrane domain-containing proteins [73,74,75]. Interestingly, Asi2 itself is a Doa10 substrate, likely due to an amphipathic helix near its N-terminus that may be exposed upon dissociation from Asi1 and Asi3 [76]. Like Doa10, the Asi complex functions in clearing membrane proteins as well as some soluble proteins that are predicted to form amphipathic helices. The currently identified substrates of the Asi complex suggest it may target misfolded ERAD-C substrates or proteins mislocalized from the ER to the INM [72,73,77]. The Asi complex is not conserved in higher eukaryotes or even other yeast strains, raising questions about whether other ligases have a comparable function in other organisms.

Another notable nuclear E3 enzyme is the heterodimeric ubiquitin ligase complex formed by the two RING proteins Slx5 and Slx8 [78,79]. The Slx5/Slx8 dimer had previously been implicated in mediating the response to DNA damage during replication [80]. The complex was subsequently characterized as an E3 that ubiquitylates substrates first modified with the small ubiquitin-like modifier protein (SUMO) [81,82,83]. The SUMO moieties of a substrate are recognized by the SUMO-interacting motifs of Slx5 for ubiquitylation by the Slx8 RING protein, in concert with the ubiquitin-conjugating enzymes Ubc4 and Ubc5 [84,85]. Due to this, Slx5/Slx8 is classified as a unique type of E3 enzyme, a SUMO-targeted ubiquitin-protein ligase (STUbL). Mutants of Slx5/Slx8 increase nuclear inclusions and accumulate high molecular weight SUMO-conjugates, indicating that SUMO may act as a regulatory factor to promote STUbL-directed protein degradation and prevent toxic accumulation of SUMO-conjugates [84,85,86]. Notably, SUMO is not always required for Slx5/Slx8-mediated substrate recognition [87,88]. The human homolog to Slx8, RNF4, functions as a homodimer for E3 ligase activity, contrary to the Slx5/Slx8 heterodimer [89]. However, RNF4 has been shown to complement deletion of both Slx5 and Slx8 in budding yeast [83].

## 4. Degradation of Cytoplasmic Substrates

Cytoplasmic protein quality control (CytoPQC) is a vital component of the UPS due to the high levels of protein synthesis that occur in this compartment. CytoPQC by the UPS can include co-translational degradation of nascent polypeptides stalled on ribosomes or degradation of newly synthesized proteins that have failed to properly fold or assemble [90]. Cytoplasmic ubiquitin ligases must distinguish targets from their highly abundant functional counterparts to ensure that these misfolded proteins are cleared efficiently and selectively, preventing their aggregation [91]. Such abnormal proteins may result from transcriptional mutations or errors in translation for newly synthesized proteins, or from external stressors in the case of previously folded proteins [1]. While proteasomal degradation represents an important component of CytoPQC, the decision between refolding a protein or targeting it for destruction is a crucial first step.

Proteasomal degradation and protein refolding both require ATP hydrolysis, but it is usually more energetically favorable for the cell to repair aberrant proteins rather than expend the energy for degradation and resynthesis of the proteins [92]. Cytosolic substrate (re)folding is mediated by the interplay of several molecular chaperone proteins. Hsp70 proteins, together with Hsp40 co-chaperones, are responsible for substrate recognition, usually through binding exposed hydrophobic and basic amino acids in unfolded or aggregated protein substrates (Figure 4) [93,94]. In yeast, the main Hsp70 proteins responsible for refolding in CytoPQC belong to the Ssa family [95]. Hsp40 co-chaperones can also bind directly to substrates and in addition stabilize interactions between Hsp70 and substrates in an ATP-dependent manner. This requires Hsp110 nucleotide-exchange factors (NEFs) to promote exchange of ADP for ATP (Figure 4) [96,97]. When substrate refolding is not possible, target proteins are ubiquitylated for subsequent degradation by the proteasome. This is particularly important for irreparably damaged proteins that could clog chaperone pathways through multiple futile attempts at refolding [91]. UPS substrates must either be recruited to E3 enzymes by chaperones, or the E3 must directly recognize the misfolded proteins. Hence, chaperones can aid in the recognition of unfolded proteins by maintaining their solubility and sometimes directly trafficking substrates to the ligases (Figure 4) [98,99].

Just as aggregates can form in the cell nucleus, misfolded cytoplasmic proteins that cannot be cleared by the UPS are sequestered into distinct compartments known as juxtanuclear quality control compartments (JUNQ) and insoluble protein deposits (IPODs) [43]. Although both compartments house aggregated misfolded proteins, JUNQ and IPODs reportedly have distinct cellular purposes. JUNQ, also referred to as CytoQ, appears to be a temporary repository for misfolded and ubiquitylated proteins that cannot yet be cleared by the UPS machinery in the cytoplasm. The proteins sequestered in JUNQ are in constant exchange with the cytosol and are thus subject to refolding when chaperone proteins become available [43]. As JUNQ is localized to the nuclear periphery, some studies have suggested an overlap between the function of JUNQ and the intranuclear nucleolar-adjacent INQ, or that they are even the same compartment [100,101]. In contrast, IPODs are perivacuolar and form even under non-stress cellular conditions [43,102]. While previous scholarship suggested IPODs contain irreparable proteins, their clearance depends on interaction with the disaggregase Hsp104, implying that their substrates may not always be terminally misfolded [102,103]. Additionally, the general autophagy factor Atg8 co-localizes with IPODs, suggesting they can be cleared by autophagy rather than by the UPS [104,105]. The relationship between JUNQ and IPODs is not well-defined, but both are important means to prevent misfolded proteins from harming the cell when chaperones and cytoplasmic ubiquitin ligases are not available for their refolding or proteolytic targeting.

### 4.1. The Ubr1 Pathway

Ubr1, another RING-type ubiquitin ligase, is an important E3 enzyme in CytoPQC. Ubr1 (mammalian UBR1) was originally identified for its role as an Arg/N-recognin, or N-terminal arginine recognition factor, in the yeast N-degron pathway [106,107]. In the N-degron pathway (previously referred to as the “N-end rule”), proteins are targeted for ubiquitylation based on the presence of destabilizing amino-acid residues at their N-termini; these N-terminal degron signals are often exposed when protein complexes dissociate in response to external stimuli or regulatory signals, or when proteins are endoproteolytically cleaved. If N-terminal degrons are shielded by chaperones such as those of the Hsp90 family, destruction through the N-degron pathway is avoided [108,109,110,111]. Ubr1 is the only Arg/N-recognin in *Saccharomyces cerevisiae* and directly binds either basic or bulky hydrophobic N-terminal residues for substrate ubiquitylation [107,112]. Ubr1 possesses binding pockets in its first 700 residues that are specific for either type of destabilizing N-terminus, as well as a third site that has been shown to bind some internal degron sequences [113]. Ubr1 functions in complex with a second ubiquitin ligase, the HECT-type Ufd4 [114,115,116]. In complex with Ubr1, Ufd4 acts as a ubiquitin ligase that specializes in ubiquitin chain elongation [111,117,118]. Although not an Arg/N-recognin, Ufd4 can bind some protein substrates just as Ubr1 can directly bind N-terminal degrons [113,119,120]. This function with Ubr1 is separate from the previously characterized role for Ufd4 in degradation of substrates containing an N-terminally fused ubiquitin moiety [121].

Several studies have also characterized Ubr1 as recognizing cytosolic misfolded proteins completely independently of the N-degron pathway. Specifically, it was shown to promote degradation of newly synthesized proteins, unfolded proteins, and thermosensitive mutant proteins [30,31,117,122,123]. Quite unlike its role in the N-degron pathway, Ubr1-mediated degradation of cytosolic misfolded substrates relies on chaperone interactions (Figure 3C). In unfolded protein CytoPQC, Ubr1 ubiquitylation requires the aid of the Hsp70-type chaperones Ssa1 and Ssa2 and the Hsp40 co-chaperones Sis1 or Ydj1 [124,125,126]. As mentioned earlier, these processes require nucleotide-exchange factors, with the Hsp110 proteins Sse1 and Fes1 implicated in Ubr1-mediated quality control [30,31,117,124,125,126]. Ssa1 and Ssa2 function with Sis1 and coimmunoprecipitate with Ubr1 and model substrates, suggesting a role in mediating Ubr1 substrate recognition. In contrast, Ydj1 does not coimmunoprecipitate with Ubr1, but instead binds the target protein and Ssa1, indicating that these chaperones likely work to keep misfolded substrates soluble prior to Ubr1 targeting [125]. Importantly, Ubr1 recognition of unfolded proteins may also occur in the nucleus despite the activity of Ubr1 in the cytoplasm (Figure 3C) [127,128,129]. This has been explained by specific trafficking of Ubr1 substrates into the nucleus for parallel ubiquitylation by Ubr1 and San1, as will be discussed further in Section 5.1.

### 4.2. The Ltn1 Pathway

Ltn1 (Rkr1, human LTN1) is a ribosome-associated RING E3 enzyme that is chiefly responsible for co-translational degradation of nascent polypeptides, working in the multi-subunit ribosome-associated quality control (RQC) complex [130,131]. The high volume of protein synthesis on ribosomes includes a fraction of abnormal nascent polypeptides that can stall the ribosome and prevent subsequent protein production. Such aberrant proteins may arise from mRNAs that lack a stop codon, for example, resulting in aberrant translation of mRNA poly(A) tails into poly-lysine sequences that cause polypeptide pausing inside the ribosome exit channel [131,132]. Nascent polypeptides that have arrested the ribosome are not yet completely folded and are targeted for degradation on the ribosome [133,134]. Ltn1 participates in a unique mode of CytoPQC that broadly targets aberrant substrates for proteasomal degradation by their behavior during translation rather than by their mature folding state [135].

Ltn1 targets polypeptides through its RQC complex interaction partner Tae2, which binds the stalled 60S ribosomal subunit after 40S subunit dissociation [136,137,138]. Tae2 binds the 60S subunit in a mutually exclusive manner with the 40S subunit; this prevents targeting of correctly translated polypeptides on 80S ribosomes and also limits re-association of released 40S subunits with stalled 60S subunits. When bound to a stalled translation complex bearing the nascent chain-bound 60S ribosomal subunit, Tae2 can associate with Ltn1, which binds the 60S subunit near the polypeptide exit tunnel and ubiquitylates lysine residues of the arrested polypeptides [136,137,138]. Ltn1 poly-ubiquitylation of substrates triggers recruitment of the ATPase Cdc48 and its cofactors Rqc1 and Vms1 for extraction of the nascent polypeptide from the ribosome and delivery to the proteasome [139,140,141,142].

If a stalled nascent polypeptide does not have an exposed lysine for Ltn1 to modify with ubiquitin, Tae2 can support Ltn1 ubiquitylation through an unusual mechanism of non-templated aminoacyl-tRNA recruitment. Tae2 specifically extends the polypeptide C-terminus through addition of Ala and Thr residues—or “CAT tails”—which forces the polypeptide through the exit tunnel until a lysine residue(s) is exposed to Ltn1 [138,143]. CATylation by Tae2 may also serve as a failsafe if Ltn1-mediated ubiquitin ligation does not occur, as CAT tail proteins can aggregate and induce a cellular stress response [144,145,146]. Additionally, Ltn1 also participates in ERAD of ribosome-associated proteins (ERAD-RA), serving as the major E3 responsible for degradation of translationally stalled proteins undergoing co-translational translocation into the ER [28,147]. Although Ltn1 is the primary ubiquitin ligase responsible for co-translational degradation, the E3 enzymes Hel2 and Not4 (mammalian ZNF598 and CNOT4, respectively) may have overlapping functions in co-translational CytoPQC and the degradation of abnormal nascent polypeptides (Table 1) [148].

### 4.3. Other Cytosolic Recognition Pathways

In the cytoplasm, additional ubiquitin ligases are known to contribute to PQC. The E3 enzyme Ubr2 (human UBR2), a paralog of Ubr1, primarily functions in the degradation of the unstable proteasome-subunit gene transcription factor Rpn4 [149]. Ubr2 interacts with the ubiquitin-conjugating enzyme Ubc2 and the adaptor protein Mub1 to form an Rpn4-recognition complex [150]. In addition to the crucial role of Ubr2 in regulating proteasome levels, it has also been implicated in the degradation of misfolded proteins. Like Ubr1, Ubr2 can recognize heat-denatured substrates in the cytosol, although it does not appear to be involved in degradation of most Ubr1 substrates [30,31,117]. The mode of recognition and the specificity of Ubr2 in targeting misfolded proteins in CytoPQC remain unknown.

Another significant ubiquitin ligase operating in CytoPQC is Hul5 (mammalian UBE3B and UBE3C), a HECT-type E3 that likely acts specifically through elongation of ubiquitin chains. Unlike most other ubiquitin ligases, Hul5 associates with the proteasome and adds to the poly-ubiquitin chains of proteasome-bound substrates; this activity opposes the deubiquitylating activity of the proteasome-associated DUB Ubp6 [151,152,153,154,155]. Elongation of proteasome-bound substrates may increase the processivity of the proteasome. In addition, Hul5 mediates degradation of many misfolded and low solubility cytoplasmic proteins, particularly those misfolded during heat shock [155]. Furthermore, Hul5 was shown to promote Cdc48 interaction with a model ERAD-M substrate, aiding in protein turnover by promoting Cdc48-mediated substrate extraction from the ER membrane and subsequently facilitating its ubiquitylation [156]. Consistent with its ubiquitin chain elongation role, mutants of Hul5 primarily affect poly-ubiquitylated but not mono-ubiquitylated substrates [154]. It is not currently known how Hul5 recognizes misfolded substrates, nor if chain elongation on these proteins creates specific types of chains or works to elongate chains initiated by particular E3 enzymes.

Despite its importance in CytoPQC, Hul5 is mainly in the nucleus but relocates to the cytoplasm upon heat shock [157]. Interestingly, its nuclear (re)import is blocked under stress conditions, consistent with its relevance to CytoPQC. Hul5 targets distinct protein substrates in unstressed and heat-stressed conditions; nevertheless, in both conditions the substrates are predominantly unfolded cytosolic proteins [157]. Hul5 likely mediates clearance of misfolded cytoplasmic proteins in response to stress conditions and may specifically recognize terminally misfolded proteins [154].

Rsp5 (human NEDD4) is another ubiquitin ligase that is active in the cytoplasm. Previous work concerning Rsp5 has focused on its functions in plasma membrane surveillance, endocytosis, and unstructured fatty acid synthesis [158,159]. In the endocytic system, Rsp5 interacts with a variety of adaptor proteins that recruit Rsp5 to various organelles and direct its ubiquitylation activity there [160]. However, recent studies indicate that Rsp5 might play an additional role in heat-induced CytoPQC that is distinct from its role at the plasma membrane. In this study, the Hsp40 co-chaperone protein Ydj1 recognizes cytosolic substrates that were misfolded during heat shock, serving as a substrate-adaptor protein for Rsp5 [161]. This leads to Rsp5 ubiquitylation and subsequent proteasomal degradation of the Ydj1-recongized substrates. Rsp5 participation in CytoPQC appears to be restricted to heat shock conditions, possibly only initiated under such stress conditions when parallel CytoPQC systems are overwhelmed by high levels of misfolded proteins [161].

## 5. Redundancy in PQC Ubiquitin Ligase Substrate Recognition

While the nucleus and cytoplasm serve as distinct sites of cellular PQC, several reports indicate that there is overlap in the clearance of proteins from these compartments. Surprisingly, many cytoplasmic substrates are transported into the nucleus for degradation, and certain degron sequences can be targeted by multiple E3 ligases [30,31,32,33]. Although the reasons for this overlap are not well understood, it is possible that such redundancies build an enhanced capacity for responding to diverse proteotoxic stresses. For example, many misfolded cytosolic targets are small proteins that may be able to passively diffuse through nuclear pores. If these proteins evade the CytoPQC machinery and accumulate in the nucleus, the ability of the nuclear degradation machinery to recognize these substrates would protect the nucleus from their potential toxic effects [34]. Stressful stimuli such as heat shock can lead to increases in misfolded proteins that accumulate in both the cytoplasm and nucleus. Analysis of nuclear E3 enzymes gene expression indicates that Doa10, San1, Asi1/2/3, Slx5 and Slx8 are all upregulated in response to heat stress, perhaps to accommodate an overflow of misfolded proteins from the cytoplasm [161,162,163,164]. However, chaperones are also crucial in misfolded protein recognition; chaperone-mediated refolding is also upregulated under heat shock, at least in the nucleus, as chaperone transcription is also increased [163,164].

Trafficking of many CytoPQC substrates into the nucleus appears to be a normal step leading to their elimination [30,31,32]. For example, mitochondrial proteins that are defective in mitochondrial import are directed to the nucleus for degradation mediated redundantly by the San1, Ubr1, and Doa10 ligases [165]. These mislocalized mitoproteins could otherwise aggregate and induce proteotoxic stress. This seems to run counter to the imperative of protecting the nucleus from misfolded proteins. However, nuclear import of PQC substrates could expedite their degradation inasmuch as proteasomes primarily localize to the nucleus [20,166]. Interestingly, in a proteomic study of putative degron sequences, it was shown that degrons recognized in both the nucleus and cytoplasm have different rates of turnover depending on the responsible ubiquitin ligase [59]. Moreover, in a comparison of identical protein substrates with the same degron but either with or without a functional NLS, the nuclear-associated versions tended to be targeted by more E3s and to get degraded faster [33].

Ubiquitin ligase redundancy might allow more rapid substrate degradation in general. For example, some protein substrates contain more than one degron, allowing them to be recognized by multiple ubiquitin ligases [56,86]. In these substrates, such as the yeast transcription factor MATα2, the presence of multiple distinct degrons may ensure rapid turnover triggered by different E3 enzymes whose activities may vary under different conditions or at different subcellular sites [167]. Another explanation for ubiquitin ligase overlap is the ability to create diverse ubiquitin chain linkages on an individual protein [168,169]. Degradation of misfolded cytoplasmic substrates has been reported to require addition of both K11- and K48-linked ubiquitin, while specific nuclear substrates required only K48-linked ubiquitin [32]. Whether this correlation holds more broadly is not yet known. As discussed in Section 3.2, Doa10 ERAD-C substrates are ubiquitylated with both linkages due to the action of two different E2 enzymes [70]. Ubiquitylation by two E3 enzymes (and thus addition of both K11- and K48-linkages) could contribute another step in the sorting of proteins between refolding and degradation fates [32]. It is also possible that the mixed K11/K48 chains produced in the cytoplasm serve to enhance the affinity of aberrant proteins for the relatively smaller population of cytoplasmic proteasomes in yeast. Chains with diverse linkages may be able to engage multiple sites on the proteasome for tighter or prolonged binding to ensure efficient degradation of the substrate [170]. By contrast, K11 chains may be dispensable for nuclear proteasomal degradation as the ubiquitin shuttle protein Dsk2 brings K48-linked target proteins directly to nuclear proteasomes [127].

### 5.1. Redundancy in Nuclear and Cytosolic Substrate Targeting

Multiple studies have contributed to our understanding of E3 enzyme-substrate targeting in yeast PQC pathways. Perhaps the most studied example is the multiplexing behavior observed between the ubiquitin ligases San1 and Ubr1 [30,31,32,33,127]. Although San1 is a nuclear enzyme and Ubr1 primarily functions in the cytoplasm, Ubr1 can also localize to the nucleus, and it ubiquitylates substrates in both compartments, independent of its role in the N-degron pathway [127]. Nuclear accumulation of Ubr1 is conserved in *S. pombe*, but not in mammalian cells [125,127,171,172]. However, some misfolded substrates of Ubr1 are also recognized by San1, but only when directed to the nucleus. In one study, a model substrate that was recognized by both San1 and Ubr1 was ubiquitylated solely by Ubr1 when a nuclear export signal was appended to the substrate to exclude it from the nucleus. Similarly, when San1 was localized to the cytoplasm instead of the nucleus, it was able to ubiquitylate the substrate, with contributions from Ubr1 [30]. The ability of nuclear San1 to recognize misfolded proteins from the cytoplasm may be limited by the molecular mass of the substrate, with larger substrates targeted for Ubr1-mediated degradation instead [173]. It was proposed that smaller Ubr1 PQC substrates could diffuse through the NPC and be recognized by San1, while larger proteins are excluded from the nucleus and are instead targeted by Ubr1 in the cytoplasm. These studies indicate that some substrates can be recognized by both E3 ligases when they are trafficked to the nucleus. Notably, some model substrates recognized by both Ubr1 and San1 are not completely stabilized in the absence of both E3s, suggesting additional ligases may contribute to their degradation [33].

Ubr1 and San1 can also behave redundantly with Doa10 [33,165,174]. Doa10 usually targets substrates with amphipathic α-helices with protein surface-exposed hydrophobic residues and is able to recognize both soluble and integral membrane proteins, as noted above [55,56]. Model substrates bearing short, helical Doa10 degrons can be recognized by Doa10 in either the cytoplasm or nucleus, consistent with its broad localization [42]. However, when other Doa10 substrates are specifically localized to the nucleus, they are also ubiquitylated by Ubr1 and San1 [33]. Interestingly, a set of non-helical model substrates bearing short repeats of hydrophobic residues at their C-termini were not recognized by Doa10 and were instead recognized by Ubr1 and San1 in both the cytoplasm and the nucleus [33]. It is possible that the absence of helicity in these model degrons prevents their recognition by Doa10, but that their high local hydrophobicity may be sufficient for Ubr1 and San1 recognition. Overall, these experiments demonstrate that short hydrophobic helical degrons can be directed to both membrane-associated and soluble ubiquitin ligases in a compartment-specific but redundant manner [33]. Ubr1 has also been implicated in ERAD where it may overlap functionally with Doa10 [128,129].

### 5.2. Chaperone-Dependent Substrate Translocation between the Nucleus and Cytoplasm

Redundancy between nuclear and cytoplasmic PQC ubiquitin ligases may be due to target proteins being transported between the compartments (Figure 4). Madura and colleagues reported that certain yeast nuclear proteins require exportin-1-mediated export to the cytoplasm for proteasomal degradation, although it is unclear which ubiquitin ligase(s) ubiquitylates these particular substrates [41,175]. Similarly, several studies have shown that chaperone-mediated nuclear import of misfolded proteins is required for their efficient clearance by various E3 ligases [30,31,32,127,174]. Such import depends on Hsp70 and Hsp90 chaperones, Hsp40 co-chaperones, and Hsp110 nucleotide-exchange factors (Figure 4), as noted above [176].

Degradation of cytoplasmic substrates by either Ubr1 or San1 requires the Hsp70 proteins Ssa1 and Ssa2, the Hsp40 proteins Ydj1 and Sis1, as well as the Hsp110 NEF Sse1 (Figure 3A,C) [31,95,127]. However, the dependence of San1- and Ubr1-mediated degradation on these factors is not uniform. Hsp70 proteins have diverse functions in the cell that include maintaining substrate solubility, facilitating nuclear transport, and promoting substrate ubiquitylation [30,31,117,177]. For both Ubr1 and San1, Ssa1, and Ssa2 are required to transport cytoplasmic substrates into the nucleus for ubiquitylation, but Ssa1 may also uniquely facilitate association of some substrates with San1 [31,95,174]. Interestingly, both pathways also require Ydj1 and Sis1 for substrate translocation to the nucleus, but the two Hsp40 co-chaperones serve slightly different functions [96,128,174]. Ydj1 suppresses misfolded substrate aggregation, but Sis1 is required for ubiquitylation of these Ubr1/San1 substrates, independent of its transport function [125]. Ydj1 and Sis1 may cooperate in these functions as high protein aggregation levels can sequester Sis1 and thus inhibit efficient nuclear import of misfolded substrates [178,179]. Conversely, while the NEF Sse1 is required for CytoPQC, it is only required for substrate nuclear import and is dispensable for degradation of NLS-containing CytoPQC substrates [127]. Unlike Ubr1, some highly insoluble San1 substrates also require Cdc48 for their targeting to the proteasome after San1-mediated ubiquitylation [180]; Cdc48 segregase activity may remove substrates from aggregates following their ubiquitylation to allow their degradation by the proteasome [90,180,181].

As discussed in Section 5.1, Ubr1 and San1 also seem to overlap somewhat with the ERAD ubiquitin ligase Doa10. This seems to also be true of their involvement with chaperone proteins, though the use of chaperones for Doa10 is likely distinct from that for Ubr1 and San1. Doa10 requires Hsp70-family chaperones for substrate degradation and several of its substrates require either Ydj1 or Sis1 as well (Figure 3B) [99,177,182]. However, Doa10 is resident at both the nuclear and cytoplasmic faces of the NE, so it is unlikely that chaperone-mediated nuclear transport is necessary to accomplish Doa10-mediated degradation, in contrast to Ubr1 and San1. This is supported by the fact that the Hsp110 protein Sse1 is entirely dispensable for substrate ubiquitylation by Doa10 [42,127]. These chaperones may either act as adaptors for Doa10-substrate association or recognize misfolded proteins and keep them soluble prior to Doa10 recognition [99,183].

Although several studies have indicated the importance of chaperones for nuclear import and San1/Ubr1-mediated degradation, it is currently unclear whether chaperone proteins utilize the classical cellular nuclear import machinery. For classical NLS-containing proteins, the nuclear transport receptor (NTR) importin-α binds the NLS, and this allows importin-α binding to the importin-β NTR [184,185,186,187]. The α/β heterodimer mediates import of the cargo protein through the NPC via transient interactions between importin-β and the disordered Phe-Gly-repeat regions of nucleoporins that form a restrictive matrix inside the NPC [188,189,190,191]. Once inside the nucleus, importin-β is released from importin-α through interaction with the active GTP-bound form of the small GTPase Ran; this triggers cargo release [192,193]. The import factors are subsequently recycled to the cytoplasm for repeated rounds of nuclear import [194,195,196].

Currently, no evidence exists to suggest that chaperones bind to these nuclear import factors directly, though they may be indirectly connected. In a yeast importin-α mutant, nuclear import defects were suppressed during heat shock and the rate of nuclear import increased [197]. Consistent with an induction of chaperone proteins during heat shock, overexpression of the Hsp70 protein Ssa1 similarly enhanced nuclear import, indicating that Ssa1 could in some way stimulate successful nuclear import [197]. Ssa1 might maintain the solubility of misfolded proteins for presentation to importin-α, or compensate for importin-α dysfunction by acting as an unconventional NTR. Interestingly, some molecular chaperones may not require importins for nuclear uptake. In one study, the Hsp70 chaperone Ssa2 was shown to transport tRNAs to the nucleus in complex with co-chaperones Ydj1 and Sis1. Importantly, this complex was able to pass through the NPC through Ssa2 interactions with the Nup116 nucleoporin, mimicking the function of importin-β [198]. These studies seem to contrast with what has been reported in metazoans. In mammalian cells, various stress conditions block nuclear import through nuclear sequestration of importin-α [199,200]. Under heat shock conditions, the mammalian Hsc70 protein accumulates in the nucleus as well; it is transported into the nucleus by a non-canonical NTR [201]. These findings suggest import of misfolded cytosolic proteins will be inhibited by heat stress, while nuclear accumulation of Hsp70 chaperones at the same time could promote NucPQC.

The Hsp70/40/110 chaperone system is part of a triage system to distinguish misfolded proteins and ensure their efficient degradation regardless of cellular compartment [96,127]. In one model, cytoplasmic misfolded substrates are recognized by Ssa1/2 and Ydj1 and kept soluble in that compartment until ubiquitylated by Ubr1 [31,95,127]. Ubiquitylated substrate then recruits Sis1, which may either assist addition of K11-linked ubiquitin by Doa10 for cytoplasmic degradation, or recruit Sse1 for nuclear transport of the substrate. Once in the nucleus, the misfolded protein can also be ubiquitylated by San1 before its ultimate proteasomal degradation [177,178,179,180,181,182,183]. Nuclear import of misfolded proteins might not be the most efficient route to their elimination. Chaperone-mediated nuclear import of aberrant proteins is slower than classical nuclear import [31], which would allow Hsp70s more time to help refold a substrate and release it before Hsp40/110-mediated nuclear import [31]. Similarly, protein refolding chaperones are also present at far higher levels than the nuclear ligase San1, possibly to outcompete nuclear degradation for folding-competent targets [164,202]. Ultimately, the dynamic network of chaperones and ubiquitin ligases appears to work in concert and often redundantly to ensure refolding or degradation of aberrant proteins in both compartments.

### 5.3. Relevance to Other Organisms

Protein quality control is critical to the well-being of cells, but many mysteries still exist regarding the underlying mechanisms. We do not have a clear structural understanding of the features of misfolded proteins that direct them to the various PQC pathways offered by the UPS. Even in yeast it has been difficult to untangle the overlapping functions of the different E3 enzymes throughout the cell. Whether the redundancy in the PQC systems observed in yeast is conserved in different organisms remains an open question but is likely. Of note, the yeast ubiquitin ligase San1 has no known mammalian homolog, so the types of degrons the San1 E3 recognizes might be targeted differently in other eukaryotes [47]. Similarly, although some studies indicate a population of yeast Ubr1 in the nucleus, Ubr1 appears to be exclusively cytoplasmic in mammalian cells [127,172]. If Ubr1 does not localize to the nucleus, this particular pathway of chaperone-dependent nuclear degradation is not expected to be conserved either. It is possible that related ubiquitin ligase networks have evolved in other eukaryotes. A notable example is the mammalian Hrd1 homolog Syvn1 (HRD1) which, in addition to sharing conserved ERAD functions, has been implicated in the degradation of soluble proteins [203,204]. This differs from yeast Hrd1 which has only been directly implicated in the clearance of ERAD-M and ERAD-L substrates [28]. Despite the general evolutionary conservation of ubiquitin ligases such as Ubr1 and Hrd1, it is striking that their detailed functions differ in such significant ways across organisms. Conversely, the mammalian ubiquitin ligase CHIP has been shown to bind Hsp70/90 chaperones and ubiquitylate chaperone-bound proteins, similar to chaperone interactions described for many yeast E3 enzymes; CHIP has no homolog in *S. cerevisiae* [205,206]. The complexity of the human UPS may explain the lack of many yeast orthologs in this and other examples—humans have an estimated 500 to 700 ubiquitin ligases, compared to roughly 100 in yeast [207].

## 6. Conclusions

Organisms rely on efficient protein quality control to protect their cells from harmful misfolded proteins and protein aggregates. When misfolded proteins cannot be refolded by molecular chaperones, they are degraded by the ubiquitin-proteasome system or selective autophagy. The degron properties that promote recognition of a given substrate by a particular E3 enzyme are still far from being fully elucidated. Several yeast ubiquitin ligases in the nuclear and cytoplasmic compartments act redundantly in the recognition and ubiquitylation of misfolded proteins, but the full range of E3 redundancy remains to be worked out. Additionally, chaperone proteins carry out various functions to mediate transport and degradation of cytoplasmic proteins in the nucleus and may also have slightly different functions when working with different E3 enzymes. The PQC crosstalk between the nucleus and cytoplasm may act as an overflow system to support an easily saturated CytoPQC machinery; alternatively, E3 ligase redundancy may simply ensure that misfolded proteins are rapidly degraded regardless of their ultimate localization. Future research efforts can be expected to bring us closer to deciphering the mechanisms that decide the fate of the vast array of differently aberrant proteins and the conditions that allow their targeting by specific ubiquitin ligases.

## Figures and Tables

**Table 1:** Known ubiquitin ligases participating in yeast protein quality control.

Ubiquitin Ligase	Known Human Homologs	Quality Control Function
Asi Complex	-	(Asi1/2/3) INM-associated degradation
Doa10	MARCHF6	ERAD-C, INM-associated degradation, N-degron degradation
Hel2	ZNF598	Histone degradation
Hrd1	SYVN1	ERAD-M, ERAD-L
Hul5	UBE3B and UBE3C	Cytoplasmic PQC, proteasome-associated ubiquitylation
Ltn1 (Rkr1)	LTN1	Co-translational degradation
Not4	CNOT4	Co-translational degradation
Rsp5	NEDD4	Nedd4 ligase, Golgi-associated degradation and heat-induced cytoplasmic PQC
San1	-	Nuclear PQC, degradation of select cytoplasmic substrates
Slx5	-	SUMO-targeted ligase, with Slx8
Slx8	RNF4	SUMO-targeted ligase, with Slx5
Tom1	HUWE1	Histone degradation
Tul1	-	Golgi-associated degradation
Ubr1	UBR1	Nuclear and cytoplasmic PQC, Arg/N recognin
Ubr2	UBR2	Cytoplasmic PQC

Table of the known ubiquitin ligases in *Saccharomyces cerevisiae* that participate in protein quality control functions. The table includes the most notable homologs in humans where known, as well as a brief description of the PQC function of a particular ubiquitin ligase.

## Data Availability

Not Applicable.
